# Rapid weight loss among elite-level judo athletes: methods and nutrition in relation to competition performance

**DOI:** 10.1080/15502783.2022.2099231

**Published:** 2022-07-13

**Authors:** Maruša Štangar, Anja Štangar, Volha Shtyrba, Blaž Cigić, Evgen Benedik

**Affiliations:** a Department of Food Science and Technology, Biotechnical Faculty, University of Ljubljana, Ljubljana, Slovenia; b Sports Unit, Ministry of Defence, Ljubljana, Slovenia; c Division of Paediatrics, University Medical Centre Ljubljana, Ljubljana, Slovenia; d Institute for Biochemistry and Molecular Genetics, Faculty of Medicine, University of Ljubljana, Ljubljana, Slovenia; e Slovenian Judo Federation, Slovenska Bistrica, Slovenia

**Keywords:** Weight cutting, diet, body mass, combat sports, success

## Abstract

**Background:**

Rapid weight loss (RWL) followed by rapid weight gain (RWG) is a regular pre-competition routine in combat sports and weightlifting. With the prevalence of these sports exceeding 20% at the 2020 Tokyo Olympics, there are limited data on RWL and RWG practices and their impact on well-being and competitive success in elite-level athletes.

**Methods:**

A total of 138 elite-level female and male judokas, 7.7% of the athletes ranked as top 150 on the International Judo Federation Senior World Ranking List (WRL), completed a survey on RWL, RWG, and the consequences of these practices.

**Results:**

Our findings showed that 96% of the respondents practice RWL. The average reduced body mass percentage was 5.8 ± 2.3%. Respondents who used either of the dehydration methods – fluid restriction, sauna suit, and/or sauna/hot bath – to reduce weight were 88%, 85%, and 76%, respectively. Furthermore, 91% of the respondents reported reduced energy as a negative consequence of RWL and 21% experienced a collapse episode during the RWL period. Respondents ranked 1–20 on the WRL experienced fewer negative consequences of RWL and RWG (*p* = 0.002) and had more dietitian and/or medical doctor support (*p* = 0.040) than lower-ranked respondents. Those who started with RWL practices before the age of 16 (38%) were ranked lower on the WRL (*p* = 0.004) and reported more negative consequences of RWL and RWG (*p* = 0.014).

**Conclusions:**

This study is the first to provide insight into the RWL practices of worldwide elite-level judokas and provides valuable information for the combat sports society, especially coaches. Proper weight management and optimal timed initiation of RWL practices in a judoka’s career may contribute to success at the elite level.

## 1. Introduction

Judo is an Olympic combat sport that originated in Japan. It is best known for its spectacular throwing techniques and also involves grappling on the ground, utilizing specialized pins, control holds, arm locks, and judo choking techniques [1]. Judo combines anaerobic and aerobic exercises. During a judo match, which is characterized by short duration, high intensity, and intermittence, the primary source of energy is anaerobic glycolysis [2]. Due to the complexity of energy demands and body mass (BM) manipulation processes before competitions, individualized dietary support is needed [3]. Similar to other combat sports, where athletes are divided into weight categories, regulation of BM is vital [4]. To gain physical advantages, most judokas compete in a weight category with a limit below their normal day-to-day BM. Therefore, rapid weight loss (RWL) is a common pre-competition practice. RWL is defined as fast weight loss in few days (up to 1 week) before weigh-in, achieved by a variety of RWL methods such as reduced food and fluid intake, restriction of individual food groups, and increased exercise with additional clothing (“sweat/sauna suits”) and sauna, both of which increase sweating. Fluid restriction and increased sweating can result in dehydration [4]. RWL is followed by rapid weight gain (RWG) after weigh-in, primarily due to water and gastrointestinal (GI) tract content loss and regain. Diet plays an important role in optimizing the RWL and regeneration period [4]. The regeneration period is attributed to the time between the official weigh-in and the start of competition the next day, which in judo must be at least 12 h [5]. The priority of regeneration is rehydration, glycogen restoration, GI comfort, and up to 5% optimal BM regain [4].

In judo, there is no “off-season”; therefore, weight cycling occurs throughout the year [6]. RWL and RWG can negatively affect athletic performance [7], mood and sleep [8–10], and GI well-being (constipation, diarrhea, and stomach pain) [11–14] and increase the risk of injury [10] and eating disorders (binge eating, anorexia, and bulimia) [4,9,14,15]. However, fighting in a lighter weight category with RWL practices may also have physical and psychological benefits [9,14,16]. RWL of about 5% has minimal detrimental effect on strength and aerobic and anaerobic capacities if athletes have at least 3 h of regeneration time. Aerobic and anaerobic performance might be impaired if athletes have less than 3 h of regeneration time [17]. GI symptoms that are common to high-level athletes [12,13] may result from altered food and fluid intake, dehydration, hyperhydration, and psychological factors, such as stress, anxiety, and/or intense physical exercise [11–13]. Extreme dehydration can cause serious health risks including fainting/collapse [18]. However, even dehydration with as little as 2% BM loss impairs motoric and cognitive abilities [19,20]. Dehydration with more than 5% BM loss worsens performance, and it is difficult to achieve euhydration within the regeneration period [3]. In 2015, the International Judo Federation (IJF) introduced random weigh-in on the morning before a competition, where an athlete’s BM must not be more than 5% above than the official maximum BM limit of their respective categories, with the aim of curbing extreme RWL practices [5].

Previous studies have provided good evidence that RWL practices can negatively affect health [7,9,10,21]. Still, ambitious judokas as well as coaches are prepared to suffer a lot of side effects for their success. Therefore, the following research questions were set for this study: how does RWL affect success and what do the BM manipulation practices of the world’s best judokas involve?

We analyzed the practices of pre-competition BM manipulation among highly trained, elite-level judokas within the top 150 positions of the IJF World Ranking List (WRL) from all continental federations. We also assessed the consequences of these practices on the competitor’s well-being and readiness. The main goal of this study was to gain insight into RWL behaviors among elite-level judokas and to assess whether it is codependent with success, which remains unanswered in previous studies [15]. To the best of our knowledge, this is the first study to include judokas from all continents worldwide at the highest senior level.

## 2. Materials and methods

The study was performed in accordance with the provisions of the Declaration of Helsinki on research involving human participants and approved by the Nutritional Research Ethics Committee of the Biotechnical Faculty, University of Ljubljana, Slovenia (No. KEP-1-7/2021). The need for written consent was waived due to exempt status. Prior to entering the survey, all participants indicated their willingness to voluntarily participate in the study and agreed for their data to be analyzed. No paradata were collected or processed.

### 2.1. The survey

This survey (Appendix) was conducted using the web-based software OneClickSurvey (https://www.1ka.si/d/en, Version 21.02.16, Faculty of Social Sciences, University of Ljubljana, Ljubljana, Slovenia). It was available in six languages: English, French, Portuguese, Spanish, Russian, and Japanese. The English version was translated into other languages by native speakers with judo background.

The survey was custom-designed for the target group, i.e. judokas in the top 150 on the WRL. The success level of the respondents was based on their WRL position at the time of the survey. It was a snapshot of the WRL position, which represents the results of the last two-year period. Questions were developed in collaboration with elite-level active athletes and coaches. The survey contained 20 questions divided into three parts: (1) general information (sex, weight category, current WRL position, continental federation, age, and formal education status); (2) questions about the RWL process (age group at which a judoka started practicing RWL (AgeRWL), day-to-day BM, height, use of different RWL methods, dietary changes, sources of information about RWL and diet, tracking caloric intake, special diets, and psychophysical consequences of RWL), and (3) questions about the regeneration/RWG process and status on competition day (diet after weigh-in and on competition day, constant ritual for food and beverage intake, and impacts of RWL followed by RWG process). RWL, a synonym for weight cutting, was defined as “fast weight loss in few days (up to 1 week) before weigh-in, achieved by food restriction, dehydration (fluid restriction and sweating), intensive exercise …“ Prior to the finalization of the survey, a pilot testing was performed, and it was tested and retested among translators, coaches, and judokas to assess their understanding of the questions.

### 2.2. Participants and data collection

This survey was available from January 7 to March 7, 2020. The inclusion criteria were to be an international judoka, at the time of the survey, ranked in the top 150 on the WRL in either of the six female or six male weight categories. Weight categories with no upper limit (+78 kg for women and +100 kg for men) were excluded as they were not relevant to the study of RWL.

Judokas were invited to participate in our study (1) by sharing a link to the survey via official social networks and also platforms of Fighting Films, the world’s leading provider of judo media and equipment, (2) through leaflets with a QR code and basic information about the survey, and (3) through a direct request at the training camps during the time of the survey (international training camp in Mittersill, Austria, and training camp in Tokyo, Japan) and the Grand Prix Tel Aviv 2020 competition.

Of all judokas who met the inclusion criteria (*N* = 1800), 257 started responding to the survey. A total of 138 valid responses were received, representing 7.7% of the target population. For further analysis, we excluded five respondents (3.6%) who did not practice RWL. The final sample size was 133 elite-level judokas including representatives of all different categories and continental federations.

### 2.3. Statistical analysis

For statistical analysis, the following three new numerical variables were created: (1) dietitian and/or medical doctor support (DDS), (2) negative consequences of RWL and RWG (RWLG_neg), and (3) positive consequences of RWL and RWG (RWLG_pos). These variables were calculated as the sum of scored answers (yes = 2; sometimes/a little = 1; and no = 0) provided by a respondent as shown in Table 1 and were used for quantitative statistical analysis. Scores were also assigned to answers regarding the athletes’ WRL position (1–20 = 1, 21–50 = 2, 51–100 = 3, and 101–150 = 4) and AgeRWL (U12 and U14 = 1, U16 = 2, U18–cadets = 3, and U21–juniors and seniors = 4). Respondents were grouped according to their sex, age, weight category, AgeRWL, and WRL position.

**Table 1.** Parameters associated with rapid weight loss and rapid weight gain.

Parameter	Definition of the parameter (sum of scored answers)	Survey question (Appendix)
DDS	Dietitian + medical doctor	Q13
RWLG_neg	Reduced energy + sleep problems + lack of motivation/determination, feeling depressed + concentration problems, confusion + GI symptoms + faint/collapse Feeling less ready for competition + problem of controlling appetite + desire to eat more + GI symptoms + being careful about morning weigh-in (5%) + insatiable thirst (competition day)	Q16 Q20
RWLG_pos	Boost in self-confidence + improved motivation/determination + improved focus feeling more ready for competition	Q16 Q20

DDS: dietitian and/or medical doctor support; RWLG_neg: negative consequences of RWL and RWG; RWLG_pos: positive consequences of RWL and RWG; GI: gastrointestinal.

The results were analyzed using IBM SPSS Statistics for Macintosh, Version 27.0 (IBM Corp., Armonk, NY). To assess the statistically significant differences (*p* ≤ 0.05) between the groups, we performed nonparametric tests: Mann–Whitney U-test for two independent groups and Kruskal–Wallis test followed by pairwise *post hoc* test using the Bonferroni correction for more than two independent groups.

## 3. Results

### 3.1. Prevalence and descriptive data

Of all 138 respondents, 133 (96%) practiced RWL. This sample (*N* = 133) was used for further analysis and included representatives from all categories and all five continental federations: European Judo Union (*N* = 109), Judo Union of Asia (*N* = 10), Pan-American Judo Confederation (*N* = 10), African Judo Union (*N* = 2), and Oceania Judo Union (*N* = 2).

The distribution of respondents according to sex, age, weight category, WRL position, and AgeRWL is shown in Table 2. Female and male judokas were grouped into three weight category groups: light-weight (44% of respondents), middle-weight (41%), and heavy-weight (15%). The highest percentage of respondents (43%) were ranked in the top 50 on the WRL. A proportion of 38% of the respondents began RWL practices before the age of 16, 38% at the age of cadets (U18, 16–17 years of age), and 24% at an older age.

**Table 2.** Descriptive data of respondents (*N* = 133).

Variable	Group	Respondents *N* (%)
Sex		
	Females	93 (69.9)
	Males	40 (30.1)
Age (years)		
	≤20	44 (33.1)
	21–25	61 (45.9)
	26–30	21 (15.8)
	>30	7 (5.3)
Female category		
Light-weight	−48 kg	23 (17.3)
	−52 kg	20 (15.0)
Middle-weight	−57 kg	20 (15.0)
	−63 kg	15 (11.3)
Heavy-weight	−70 kg	10 (7.5)
	−78 kg	5 (3.8)
Male category		
Light-weight	−60 kg	10 (7.5)
	−66 kg	6 (4.5)
Middle-weight	−73 kg	7 (5.3)
	−81 kg	12 (9.0)
Heavy-weight	−90 kg	2 (1.5)
	−100 kg	3 (2.3)
WRL position		
	1–20	21 (15.8)
	21–50	36 (27.1)
	51–100	27 (20.3)
	101–150	49 (36.8)
AgeRWL		
	U12	3 (2.3)
	U14	17 (12.8)
	U16	30 (22.6)
	U18–cadets	51 (38.3)
	U21–juniors	25 (18.8)
	seniors	7 (5.3)

WRL: World Ranking List; RWL: rapid weight loss; AgeRWL: age group at which a judoka started practicing RWL; U: under.

Competitors rely on different sources of information about RWL and nutrition (Figure S1). Apart from their own experiences (98%), they obtain information from other judokas (80%), coaches (75%), Internet-based sources (73%), books (66%), dietitians (64%), and medical doctors (29%). Twenty individuals (15%) follow at least one of the special diets including gluten-free (9%), vegetarian (6%), lactose-free (6%), keto/low-carbohydrate, high-fat (LCHF; 4%), and vegan (2%). A proportion of 18% of respondents tracked their daily caloric intake regularly, while 40% of respondents did it sometimes. Regarding after weigh-in habits, almost all (94%) the respondents had consistent ritual of which 52% had very strict eating and drinking habits (Table S1).

### 3.2. Reduced body mass percentage and methods of rapid weight loss

**Figure 1.**
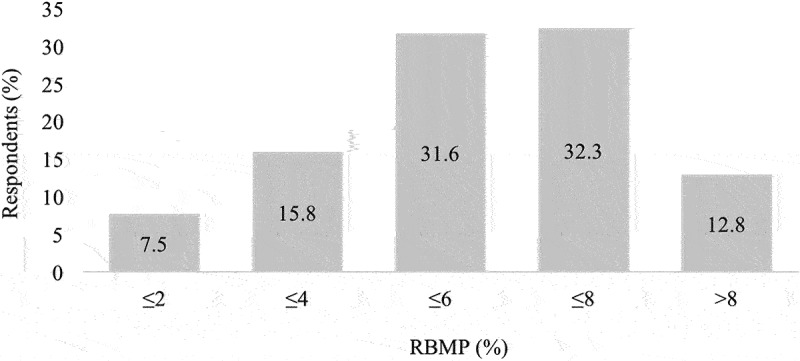
Frequency distribution of respondents according to reduced body mass percentage (*N* = 133).

Reduced body mass percentage (RBMP) was calculated as the difference between a normal day-to-day BM and the category limit divided by the normal day-to-day BM. The average RBMP was 5.8 ± 2.3%, the highest RBMP was 13.0%, and the lowest was 0.7%. The frequency distribution is shown in Figure 1.

**Figure 2.**
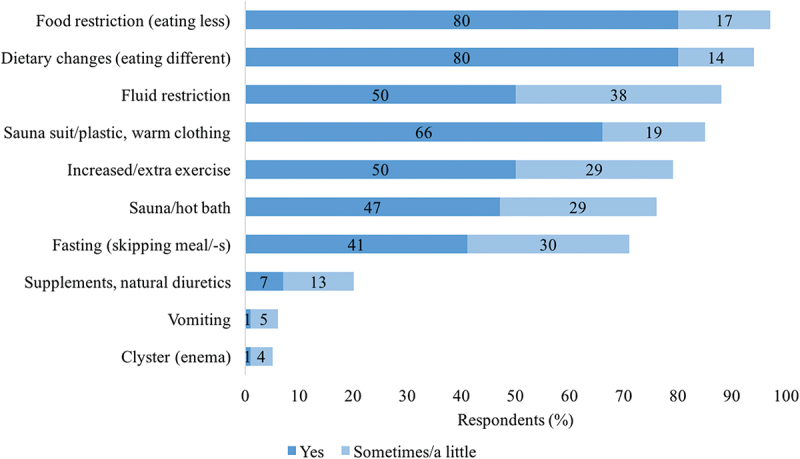
Rapid weight loss methods (*N* = 133).

The frequency of various RWL methods is shown in Figure 2. Food and fluid restrictions as well as dietary changes were the three most used RWL methods. Active (sauna suit/plastic and warm clothing) and passive (sauna/hot bath) sweating methods were also widely used by more than 75% of the respondents. Fasting or skipping meals was practiced by more than two-thirds of the respondents, while the use of supplements for body water management and/or natural diuretics was not common (20%). Some individuals used vomiting (6%) and clysters/enemas (5%) for BM reduction.

**Figure 3.**
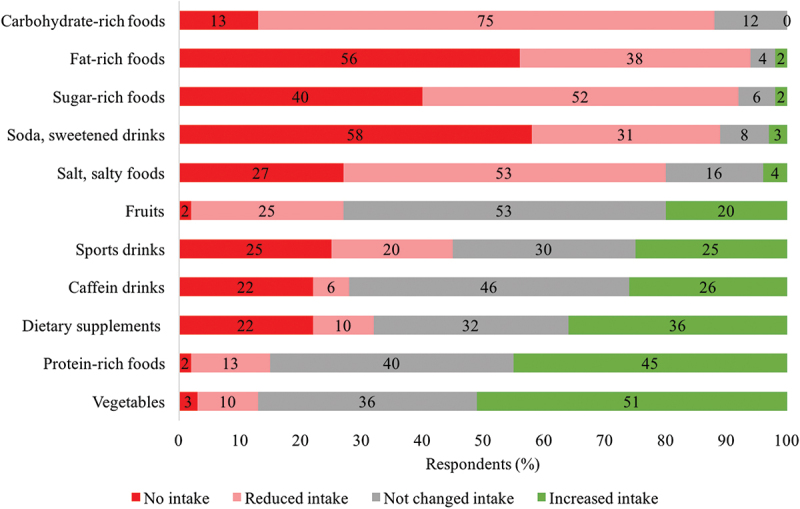
Dietary changes in the rapid weight loss period (*N* = 128).

For 94% of the respondents’ dietary changes were part of their RWL regime, making dietary changes the second most used method (Figure 2). The specific dietary changes, considering the entire diet period of about one week before competition (Figure 3), were based on reducing fat-rich and carbohydrate-rich foods. A low-salt diet was also common. On the other hand, most respondents increased the consumption of vegetables and protein-rich foods. The least changes were in the intake of fruits and caffeinated beverages.

**Figure 4.**
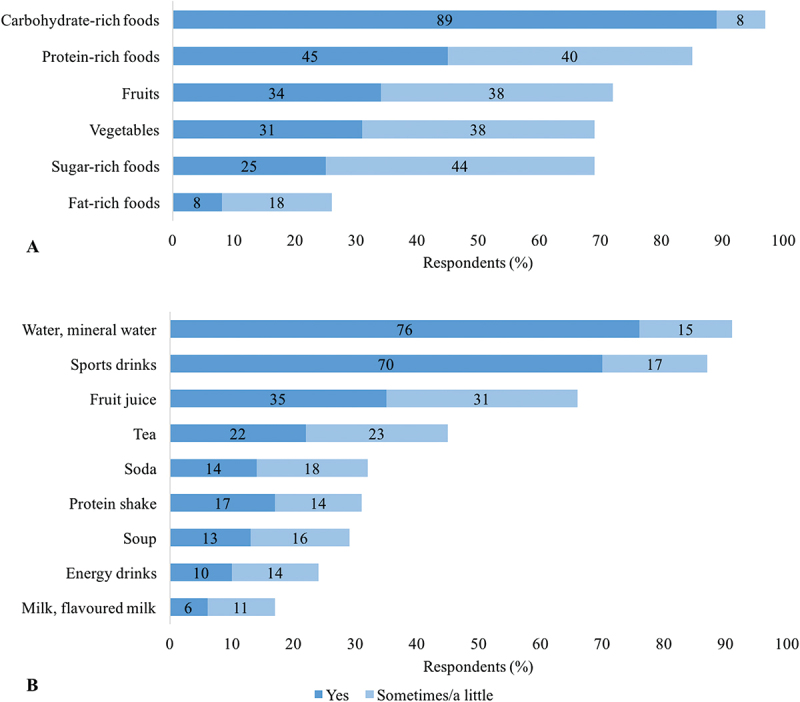
Food (A) and beverage (B) choices in the rapid weight gain period (*N* = 133).

Immediately after weigh-in, all respondents began to regenerate, first by drinking. Food and beverage choices after weigh-in (Figure 4) indicated some common practices. Respondents drank mostly water/mineral water and sports drinks. Regeneration meals mainly included carbohydrate-rich foods (89% yes and 8% sometimes/a little) and protein-rich foods (45% yes and 40% sometimes/a little), while fat-rich foods were rarely consumed after weigh-in (8% yes and 18% sometimes/a little).

### 3.3. Impacts of rapid weight loss and rapid weight gain processes

**Figure 5.**
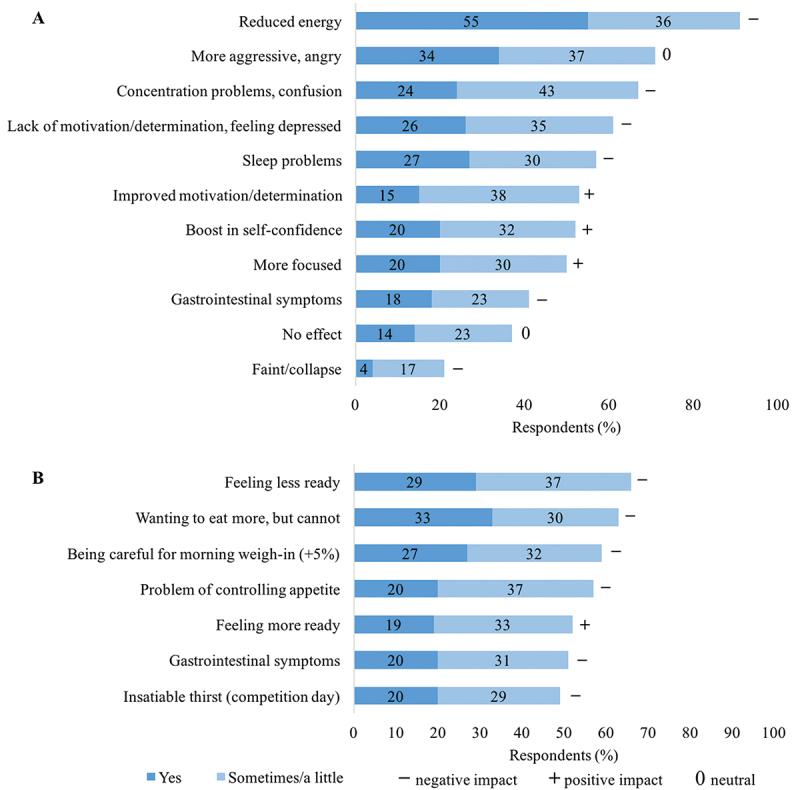
Physiological and psychological consequences of (a) rapid weight loss and (b) rapid weight gain (*N* = 133).

The breakdown of the negative as well as some positive impacts of RWL is shown in Figure 5(a). The most common (91%) negative consequence of RWL was the feeling of “reduced energy,” followed by concentration problems/confusion, lack of motivation/determination, or “feeling depressed,” sleep problems, GI symptoms, and even faint/collapse (21%). Approximately half of all the respondents perceived RWL to be beneficial for improved concentration/determination, increased self-confidence, and better focus. Increased aggression/anger was the second most reported consequence (71%). Only 14% of the respondents experienced no consequences of RWL.

Figure 5(b) illustrates the respondents’ agreement or disagreement with the given statements about RWG following RWL, which may have a positive or a negative impact on the competition readiness. However, the negative impacts were more common. The statements were as follows: After the weigh-in, judokas would like to eat more, but they cannot (63%); they have to be careful to stay within the allowed 5% of BM increase until the morning weigh-in (59%); they have problems controlling their appetite and eat too much (57%); they get GI symptoms (51%); and they still feel insatiable thirst on the competition day (49%).

### 3.4. Differences of weight management parameters and success between respondents

The present study examined the differences between respondents’ sex, category, age, AgeRWL, and the WRL position in relation to RBMP, RWLG_neg, RWLG_pos, DDS, AgeRWL, and the WRL position (Table 3). Males and females did not differ in any parameter. The following differences were found: (1) RBMP among respondents of different categories; (2) RWLG_neg among respondents of different ages, AgeRWLs, and WRL positions; (3) DDS among respondents of different WRL positions; (4) AgeRWL among respondents of different ages and WRL positions; and (5) WRL position among respondents of different ages and AgeRWLs.

**Table 3.** Differences between respondents in selected variables analyzed using nonparametric Mann–Whitney *U*-test between two groups and Kruskal–Wallis test between three or more groups (*N* = 133).

		RBMP	RWLG_neg	RWLG_pos	DDS	AgeRWL	WRL position
Sex							
	Mean ranks						
Female (*N* = 93)		64.87	67.38	65.56	69.48	63.84	
Male (*N* = 40)		71.95	66.11	70.35	61.24	74.35	
	*p*-value	NS	NS	NS	NS	NS	
Category							
	Mean ranks						
Light-weight (*N* = 59)		76.26	66.50	62.47	71.58	64.32	
Middle-weight (*N* = 54)		62.20	62.66	73.16	61.81	74.71	
Heavy-weight (*N* = 20)		52.63	80.20	63.73	67.53	54.08	
	*p*-value	0.030	NS	NS	NS	NS	
Age							
	Mean ranks						
≤20 (*N* = 44)		60.92	78.35	72.30	66.65	52.94	84.42
21–25 (*N* = 61)		75.59	66.60	63.62	63.70	70.31	62.72
≥26 (*N* = 28)		57.84	50.04	66.04	74.73	81.88	48.95
	*p-*value	NS	0.010	NS	NS	0.003	0.000
AgeRWL groups							
	Mean ranks						
≤U16 (*N* = 50)		59.20	77.51	64.53	66.58		79.03
≥U18 (*N* = 83)		72.70	60.67	68.49	67.25		59.75
	*p*-value	NS	0.014	NS	NS		0.004
WRL groups							
	Mean ranks						
1–20 (*N* = 21)		62.62	43.19	64.88	82.24	80.93	
21–50 (*N* = 36)		77.94	70.96	68.54	73.50	72.14	
51–100 (*N* = 27)		66.54	57.81	61.70	55.04	72.02	
101–150 (*N* = 49)		61.09	79.36	69.69	62.29	54.49	
	*p*-value	NS	0.002	NS	0.041	0.020	

NS: not significant. Bold numbers indicate statistically significant differences (*p* < 0.05). RBMP: reduced body mass percentage; RWLG_neg: negative impacts of rapid weight loss (RWL) and rapid weight gain (RWG); RWLG_pos: positive impacts of RWL and RWG; DDS: dietitian and/or medical doctor support; AgeRWL: age group at which a judoka started practicing RWL; WRL: World Ranking List.

RWLG_neg differed between age, AgeRWL, and WRL groups, whereas RWLG_pos were similar among all observed groups.

RBMP decreases with higher weight categories but was not associated with RWLG_neg or RWLG_pos, nor with the WRL position.

Respondents who were up to 20 years of age experienced more RWLG_neg and started with RWL practices earlier in their career compared to respondents aged 26 or older (*p* = 0.007 and *p* = 0.004, respectively; Figure S2). The respondents in this age group were also lower ranked on the WRL compared to the rank of the older respondents (21–25 years, *p* = 0.009; ≥26 years, *p* = 0.000; Figure S2).

Higher WRL position correlated to the later start of RWL practices in career. The respondents who started with RWL younger than 16 years were significantly lower ranked on the WRL (*p* = 0.004; Table 3). The respondents who were in the highest success group (1–20 WRL) had significantly higher AgeRWL compared to those in the lowest success group (101–150 WRL, *p* = 0.036, Figure S2).

Both AgeRWL and the WRL positions were also related to RWLG_neg. Respondents who started with RWL later in their career (≥U18) and those in the highest success group (1–20 WRL) experienced less RWLG_neg compared to their counterparts (*p* = 0.014, *p* = 0.002, respectively).

There were differences in DDS between the success groups (Table 3), where the most successful respondents (1–20 WRL) received significantly more DDS compared to others (*p* = 0.040; Table S3).

## 4. Discussion

This study stands out mostly because of the sample of only the world’s best judokas from all continental federations and weight categories. The distribution of continental federations was heterogeneous, with the majority of respondents from the European Judo Union. However, members of the European Judo Union account for more than half of the competitors registered at the IJF. We recruited 7.7% of all judokas ranked in the top 150 on the WRL (10.8% for female and 4.6% for male) and 9.5% of all judokas ranked in the top 50 on the WRL in the six female and male weight categories. The large sample size and novel survey design of this study provide unique and relevant insight into RWL and RWG practices and dietary changes in the pre-competition and competition periods among the world’s elite-level judokas, which is an important research area lacking relevant data [15].

### 4.1. Body mass reduction

Almost all respondents (96%) practiced RWL, which appears to be a regular pre-competition routine for elite-level judokas. Previously, the prevalence of RWL ranges from 40% to 93% regardless of the type of combat sport, age, sex, and competition level [15,16,22–26]. The lower prevalence of RWL reported in previous studies was probably due to the responses from younger/lower-level judokas, fewer judokas, and/or judokas from only one country.

The average RBMP of 5.8% was within the range of 2–10% reported by previous studies (mostly around 5%) that included judo and other Olympic combat sports athletes [3,7,10,23,24]. It was also within the range of the optimal RBMP according to the guidelines that BM should be 5–6% above an athlete’s weight category one week before competition [3]. If normal day-to-day BM is higher than that, gradual dieting for more than one week ahead of the competition and moving in an upper weight category are options to be considered [3]. On the other hand, day-to-day BM below or very little above limit is also not optimal because it could cause physical disadvantage competing against a heavier opponent [27].

Athletes in lighter categories reduced relatively more BM than those in heavier categories, but they did not differ in RWLG_neg or any other RWL parameter (Table 3). Because RWL practices rely on body water manipulation and minimal reliance on body fat loss, individuals in light-weight categories with leaner body composition [28] seem to have relatively greater capacity for BM reduction compared to individuals in heavy-weight categories.

### 4.2. Rapid weight loss and world ranking list position

From previous studies, the differences in weight-cutting utilization between different competitive levels [15] and the impact of RWL on performance [10] remain unclear. However, the present results provide sufficient evidence that RWL and success are interdependent.

The most outstanding finding of the current study is that the top 20 ranked judokas had less RWLG_neg compared to lower-ranked respondents (*p* = 0.002; Table S3). This implies that more successful competitors are able to deal with the pre-competition weight management better, which supports the previous hypotheses by Barley et al. [15]. We observed no difference in RBMP between the WRL success groups (Table 3), which indicates that a good approach toward RWL is more important for success than RBMP itself.

There were differences in DDS between the success groups (Table 3), which was higher in the top 20 ranked judokas compared to those ranked 21–150 (*p* = 0.040; Table S3). The present study findings along with those reported in previous studies show that higher-level competitors compared to lower-level competitors have more support from dietitians: 86% of respondents ranked in the top 20 on the WRL and 60% of those ranked 21–150 on the WRL obtained information about RWL and diet from dietitians. Lower-level judokas included in previous studies received low dietitian support: among regional to international level Brazilian and national to international Australian judokas, it was 19–28% [22,23] and 23% [24], respectively.

Considering the above, more successful judokas manage RWL with less negative side effects and have higher expert support compared to less successful judokas. The question still remains whether negative consequences and expert support are codependent and whether success is a cause or consequence of both factors.

In the lowest success group (101–150 WRL), judokas start RWL earlier (*p* = 0.036) in their career than that observed in the highest success group (1–20 WRL; Figure S2). The difference in AgeRWL among the success groups was retained when an age-weighted analysis was performed. These results indicated that an early start of RWL could negatively affect the success of competitors.

### 4.3. Negative outcome of early start of rapid weight loss in the judo career

The most common AgeRWL was the age group of cadets (U18; Table 2) when the first official IJF competitions and continental and world championships take place. Judokas who started practicing RWL as early as U12, U14, or U16 were mostly younger, were lower ranked, and had more RWLG_neg compared to those observed in judokas who started RWL at U18 or later (Table 3). The association between the WRL position and RWLG_neg was retained when an age-weighted analysis was performed. Thus, an early start of RWL combined with negative consequences could be a selective and success-related factor in judo that may prevent a successful long-term career. Most importantly, early start of RWL is inappropriate because of the health risks (impaired nutritional status, decreased physical performance, impaired growth and development, long-term problems related to RWL, etc.) [7,9,10,21]. Our study results together with the literature provide good evidence that starting RWL earlier in an athlete’s career negatively affects health and long-term success. This is an important message, especially for coaches of young athletes.

### 4.4. Rapid weight loss methods and dietary approaches in the rapid weight loss and rapid weight gain periods

In our study, there was a higher use (76–88%) of dehydration methods (Figure 2) including fluid restriction, sauna suit, and sauna/hot bath compared to that reported in previous studies (23–75%) [22–24,26]. A detailed comparison of the prevalence of RWL methods is presented in Table S2. The respondents in these studies include lower-level judokas, suggesting that dehydration methods are more likely to be used by high-level athletes. These methods can lead to severe dehydration [3,4]. The high prevalence of the use of dehydration methods should attract the attention of coaches, doctors, dietitians, and combat sport athletes to ensure that these methods, which could lead to health problems and decreased performance [19,20], are not overused.

The nutritional approach of most respondents appears to be appropriate. Carbohydrate intake was reduced during RWL (Figure 3), which promotes depletion of hydrated glycogen and causes effective RWL [3]. After weigh-in, glycogen stores are optimally replenished by carbohydrate-rich foods and moderate protein consumption [3]. Such practice was reported from most respondents (Figure 4). In the RWL period, the high protein intake of respondents is appropriate because it maximizes the maintenance of muscle mass and strength, improves regeneration, and reduces muscle microdamage [29–31]. Fats were avoided in the RWL period, as well as after weigh-in, and during the competition, which contributes to energy deficit in the RWL period and to easier digestion and reduction of the possibility of GI symptoms [3]. Higher consumption of vegetables (Figure 3), which are low in energy and high in nutritional value, is appropriate up to 3 days before weigh-in. Subsequently, fibers should be avoided to minimize GI content and avoid GI symptoms [3]. Reducing salt seems to be a common RWL strategy (Figure 3), but there are conflicting data on sufficient salt intake [24,29] and requirements for increased sweating [4,32]. There were similar macronutrient intakes with big reduction of carbohydrate-rich and fat-rich foods while maintaining the proteins in their diets in a study that analyzed 7-day food records before competition of senior national team Polish judokas (15 women and 15 men) [29]. They also reported low dietary fiber intake in the pre-competition week. Although our respondents’ intake of vegetables (high in dietary fiber) was mostly increased or unchanged (Figure 3), this survey does not provide data on actual dietary fiber intakes. Therefore, an appropriate comparison is not possible.

Our results on dietary changes for RWL (Figure 3) and RWG (Figure 4) provide a good, but not detailed, picture of the nutrition status of elite-level judokas in the RWL and RWG periods. Therefore, the nutrition of judokas/combat sport athletes, which is determined by weight cycling, requires further research.

### 4.5. Negative and positive impacts of the rapid weight loss and rapid weight gain processes

The results in this study (Figure 5) are consistent with that of previous studies that reported an increase in tension, anger, and fatigue and a decrease in vigor among athletes who practiced RWL [8,9,11,33]. Reduced energy, the most commonly reported negative impact in this study (Figure 5(a)), may be due to reduced glycogen stores [34]. Increased anger/aggression was a very common effect of RWL; however, with the athletes’ success as the main goal, increased anger/aggression should not be considered negative. Particularly, when there is a lack of strength and energy, increased anger/aggression is helpful in fighting or training, which requires determination and sharpness of movements [35]. In general, the negative effects of RWL predominate (Figure 5(a)). No significant differences in RWLG_pos were found between the WRL groups (Table 3). Nevertheless, increased focus and confidence might play an important role in the preparation for a competition [16,24]. There was an alarmingly high prevalence of fainting/collapse during the RWL period (21%; Figure 5(a)). Furthermore, concentration problems/confusion, fainting/collapse, and the feeling of insatiable thirst on the competition day are possible consequences of failure to achieve euhydration, which could be caused by excessive dehydration and/or inappropriate rehydration strategies (fluid quantity, distribution, and/or electrolyte content) [18,19]; this was beyond the scope of our study.

Almost half of the respondents confirmed GI discomfort (Figure 5) during the RWL period (41%) and slightly more reported GI discomfort after weigh-in and in the competition (51%). Dietary changes during RWL and excessive intake of carbohydrate-rich foods after weigh-in occurring together with psychological stress could contribute to worsening of the GI well-being. Problems related to GI well-being, desiring to eat more and controlling appetite, were common despite the near-constant ritual of eating and drinking after weigh-in.

### 4.6. Limitations

An anonymous online-distributed survey was conducted; therefore, the WRL ranking of the respondents could not be verified. We only obtained self-reported data, and no measurements of biochemical parameters were performed. Data on the consequences of RWL and RWG were obtained based on competitors’ perceived condition, which is subjective, but important for an athlete’s performance. No long-term consequences were investigated. The questions were closed-ended; therefore, other methods, nutrition changes/choices, information sources, and/or consequences of RWL and RWG might not have been included. For better nutrition analysis that would provide information about the timing and quantity of nutrient ingestion, a 7-day weighted food diary or similar methodology would be needed. For a large sample of international elite-level athletes, obtaining body status measurements and weighted food diaries would be challenging but worth addressing in future studies.

## 5. Conclusions

n the current study, we conducted a survey among world’s elite-level judokas regarding their RWL and RWG practices and the consequences of their practices on their well-being and competitive success. Data obtained from 7.7% of judokas ranked in the top 150 positions on the WRL revealed that the vast majority (96%) practiced RWL with an average RBMP of 5.8 ± 2.3%. Respondents used food restriction (97%) as a main dietary method and sauna suit (85%) as a main non-dietary method during RWL. In general, respondents had appropriate dietary approaches toward RWL and RWG. The highest ranked judokas on the WRL had fewer RWLG_neg, and AgeRWL was older and had more DDS. Good weight management and optimal timed initiation of RWL practices in a judoka’s career contribute to success at the elite-level. Coaches and athletes striving to achieve the best success should therefore optimize RWL and RWG as well. Although this study recruited only judokas, there are similar RWL and RWG practices in other combat sports; hence, all combat sport coaches and athletes can benefit from the present findings.

## Supplementary Material

Supplemental Material

Supplemental Material

## Appendices

- The survey. Weight cutting and after weigh-in habits among judokas.
- Figure S1. Sources used to obtain information about rapid weight loss (RWL) and nutrition (*N* = 133).
- Figure S2. Differences between age groups in relation to (A) negative consequences of rapid weight loss and rapid weight gain (RWLG_neg), (B) age group at which a judoka started practicing RWL (AgeRWL), (C) World Ranking List (WRL) position, (D) differences between the WRL groups in relation to RWLG_neg, and (E) AgeRWL analyzed with all pairwise *post hoc* test after Kruskal–Wallis test (*N* = 133).
- Table S1. Specific characteristics of the sample (*N* = 133).
- Table S2. Literature comparison of rapid weight loss methods.
- Table S3. Differences in negative consequences of rapid weight loss and rapid weight gain and dietitian and/or medical doctor support between the 1–20 and 21–150 WRL groups analyzed with nonparametric Mann–Whitney *U*-test (*N* = 133).
