# Supplementary figures and images for: Rapid weight loss among elite-level judo athletes: methods and nutrition in relation to competition performance
Source: J Int Soc Sports Nutr. 2022 Jul 13;19(1):380–96. doi: 10.1080/15502783.2022.2099231 (PMC9291696; doi:10.1080/15502783.2022.2099231)

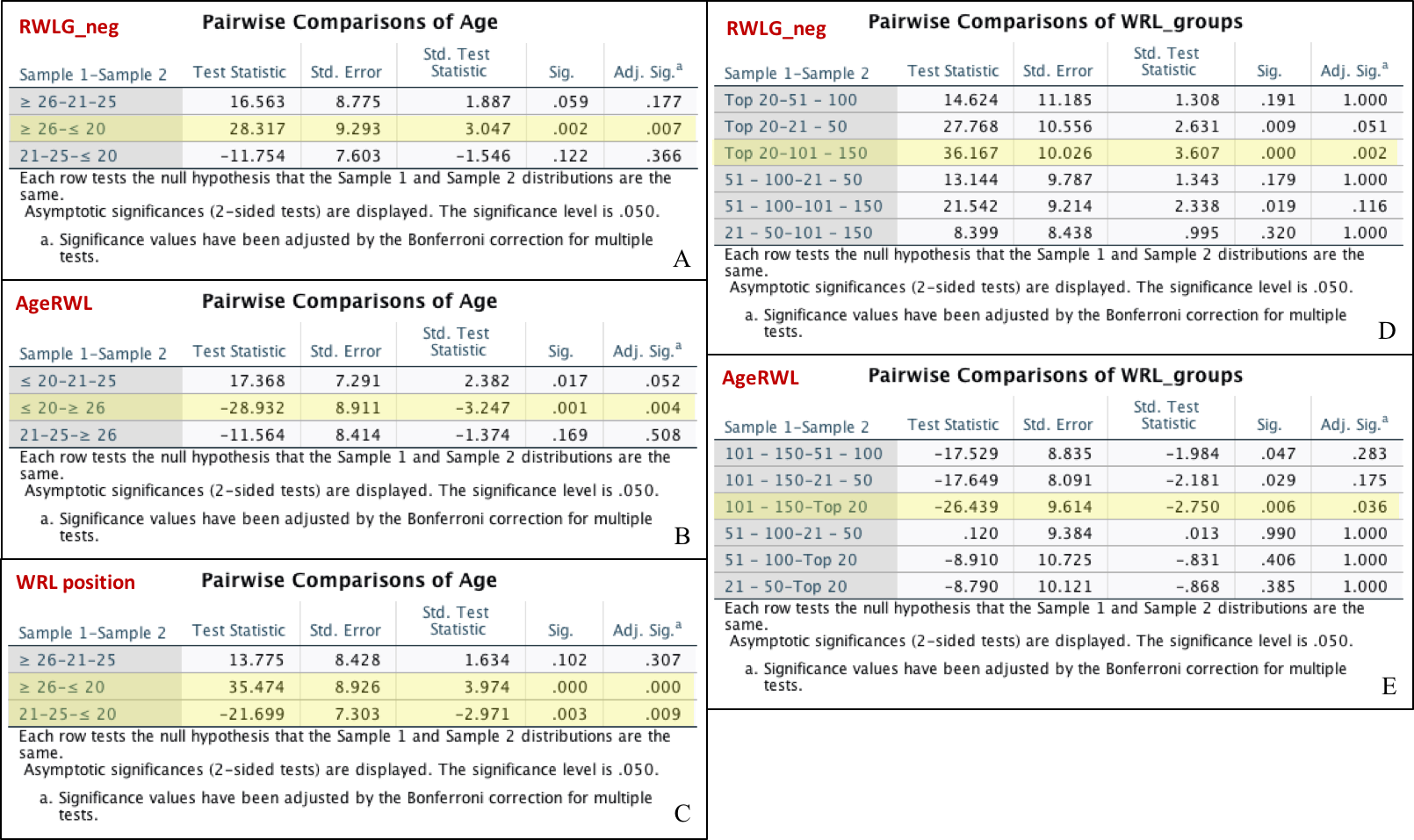

Supplement: Supplemental Material [file RSSN_A_2099231_SM7113.tiff]

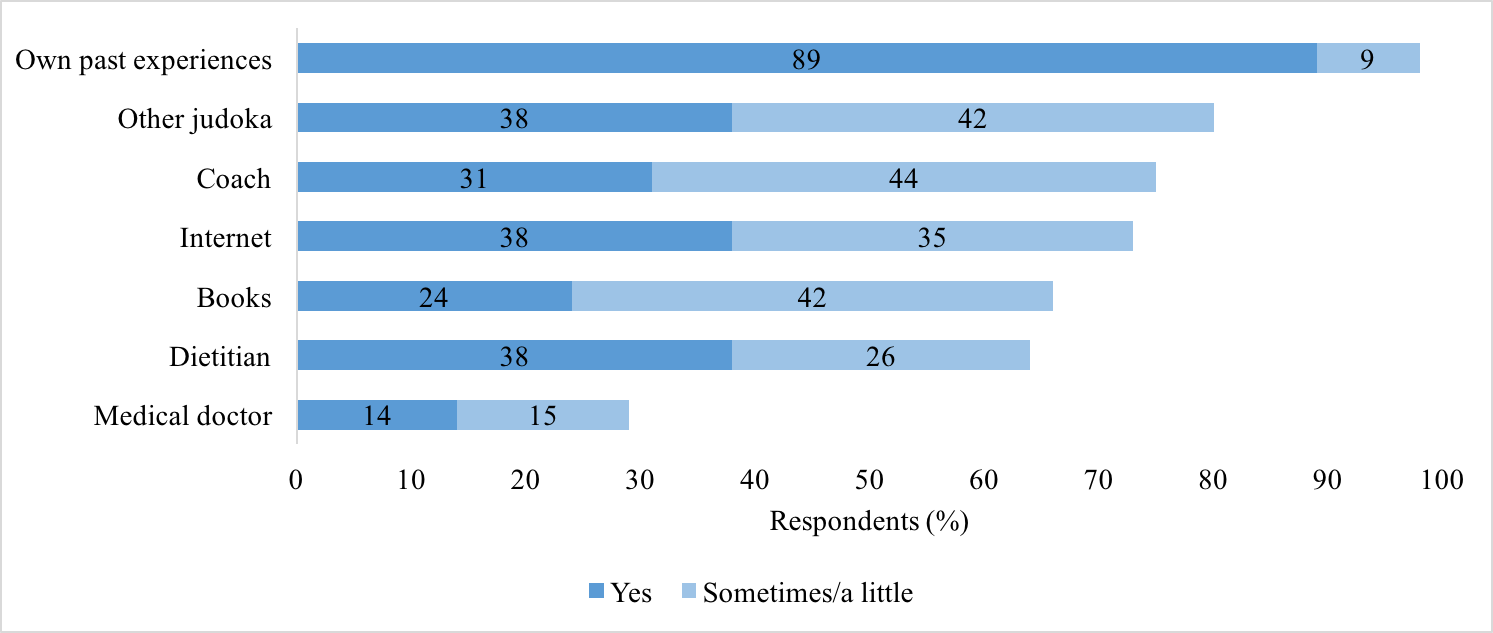

Supplement: Supplemental Material [file RSSN_A_2099231_SM7108.tiff]
